# Cardio-Oncology: mechanisms of cardiovascular toxicity

**DOI:** 10.12688/f1000research.12598.1

**Published:** 2018-01-25

**Authors:** Timothy M. Markman, Maurie Markman

**Affiliations:** 1 Department of Medicine, Cardiovascular Division, Hospital of the University of Pennsylvania, University of Pennsylvania, Philadelphia, Pennsylvania, USA; 2 Cancer Treatment Centers of America at Eastern Regional Medical Center, Philadelphia, Pennsylvania, USA

**Keywords:** cardiovascular toxicity, cardio-oncology, anthracyclines, HER2/ErbB2 inhibitors, immune checkpoint inhibitors

## Abstract

The therapeutic options available to treat a wide range of malignancies are rapidly increasing. At the same time, the population being treated is aging with more cardiovascular risk factors, comorbid conditions, and associated poor cardiac reserve. Both traditional chemotherapeutic agents (for example, anthracyclines) and newer therapies (for example, targeted tyrosine kinase inhibitors and immune checkpoint inhibitors) have demonstrated profound cardiovascular toxicities. It is important to understand the mechanisms of these toxicities to establish strategies for the prevention and management of complications—arrhythmias, heart failure, and even death. In the first of this two-part review series, we focus on what is known and hypothesized about the mechanisms of cardiovascular toxicity from anthracyclines, HER2/ErbB2 inhibitors, immune checkpoint inhibitors, and vascular endothelial growth factor inhibitors.

## Introduction

Cardiovascular disease and cancer remain leading causes of mortality in the United States. Given the rapid expansion in the number of therapeutic options for cancer, the development of cardiovascular disease in these patients presents a growing challenge for both oncologists and cardiologists. Overlapping risk factors for cancer and cardiovascular disease, especially tobacco use and advanced age, play a significant role in this association ^1^. Additionally, improved survival has helped to highlight the cardiac toxicity that results from antineoplastic therapy as patients are living long enough to experience adverse effects from these complications. This has been especially true in pediatric cancer survivors who have demonstrated increased rates of cardiovascular disease several decades after therapy ^2– 4^.

Antineoplastic agents from multiple classes affect cardiac tissue and result in myocardial cellular damage, ultimately leading to a range of clinically important effects, including electrophysiological abnormalities, symptomatic heart failure (HF), and even death. The rate of dangerous ventricular arrhythmias may increase by 10-fold in patients with cancer, and chemotherapy-induced cardiomyopathy has been reported in 1–5% of cancer survivors and is substantially higher in certain populations ^5– 7^. These toxicities represent a limiting factor in the therapy of several otherwise-treatable neoplasms, and an aging population with impaired cardiac reserve may be even more susceptible to these effects ^8, 9^. In this review, we explore both established and theoretical mechanisms of cardiac toxicity contributing to cardiovascular disease from several important classes of antineoplastic therapy.

### Anthracyclines

Anthracycline-induced cardiomyopathy was the earliest-described form of cardiotoxicity from chemotherapy. Accordingly, the mechanism has been the most thoroughly investigated and represents the broad effects and toxicities of traditional chemotherapeutic agents. This type of cardiotoxicity causes cardiomyocyte death and traditionally has been referred to as type I ^10, 11^. Anthracyclines are highly effective agents, but this toxicity, which is dose-dependent, limits their use. Cumulative anthracycline dose is capped at less than 500 mg/m ^2^ on the basis of evidence that is largely from retrospective analyses from clinical trials in adults and that suggests the rates of incidence of HF due to doxorubicin are 1.7% with a cumulative dose of 300 mg/m ^2^ and 4.7% with a cumulative dose of 400 mg/m ^2^ and increase rapidly to 15.7% at 500 mg/m ^2^ and 48% at 650 mg/m ^2^ ^12^. Cumulative doses in patients with pre-existing cardiac dysfunction can be more challenging to determine.

Anthracycline toxicity can be categorized as acute, early-onset chronic progressive, or late-onset chronic progressive ^13^. Acute toxicity is rare and occurs within hours or days of infusion and is generally reversible. This type of cardiotoxicity is not predictive of future HF and represents a small minority (1%) of cases. Early-onset chronic progressive toxicity represents the majority of cases of anthracycline cardiotoxicity and occurs within one year of therapy, whereas late onset is uncommon and occurs after one year of therapy ^14^. Although these manifestations may not occur for several years after completion of therapy, evidence from biopsy studies suggests that the initial insult occurs from the onset of therapy ^15^. Accordingly, several techniques in imaging and electrocardiographic monitoring are under investigation in order to make an early determination of which patients are experiencing cardiac toxicity ^16, 17^.

The therapeutic mechanism of action of anthracyclines involves the interruption of replicating cells through intercalation with DNA as well as the inhibition of topoisomerase II. The mechanisms of cardiac toxicity have not been conclusively established but are widely accepted to relate to multiple pathways of oxidative stress through the formation of reactive oxygen species. Interaction occurs both with topoisomerase IIα, which is overexpressed in malignant cells and is one of the therapeutic targets, and with topoisomerase IIβ, which is expressed in cardiomyocytes. Several mechanisms of the effect on topoisomerase II have been proposed, including interactions with the GTPase Rac1 and peroxisome proliferator-activated receptor γ coactivator-1α ^18– 20^. Furthermore, it seems that anthracyclines may directly impair the proliferation of cardiac progenitor cells, which impair recovery from physiologic and pathologic stressors and inhibit pro-survival signaling pathways of ErbB and NRG-1 ^21– 24^. Based on these proposed mechanisms of toxicity, many therapies—both novel and those with existing use in the treatment of cardiovascular conditions—have been considered to prevent or reverse anthracycline-induced cardiotoxicity. These agents include common cardiac medications such as 3-hydroxy-3-methylglutaryl-coenzyme A (HMG-CoA) reductase inhibitors (or statins), β-blockers, or angiotensin-converting enzyme (ACE) inhibitors and novel agents such as dexrazoxane. Their use and the evidence behind them will be discussed in the second part of this review series.

### HER2/ErbB2 inhibitors

Although anthracyclines dominated the literature and clinical concern for cardiac toxicity of antineoplastic therapy for many years, the now-widespread use of trastuzumab—a monoclonal antibody directed against HER2/ErbB2 receptors—for the treatment of breast cancers that overexpressed HER2/ErbB2 revealed an overlapping but distinct phenotype of cardiac toxicity ^25^. Trastuzumab fits into a category of targeted antineoplastic therapy that, as opposed to traditional chemotherapeutic agents (including anthracyclines), has a generally more favorable safety profile. Nevertheless, trastuzumab causes reduced cardiac function (specifically reduction in left ventricular systolic function) that is clinically similar to that caused by anthracyclines. The mechanism, however, is unique and appears to result in non-dose-dependent cardiomyocyte dysfunction rather than the cell death caused by anthracyclines ^26^. Supporting this distinction, endomyocardial biopsies from patients with trastuzumab cardiac toxicity do not demonstrate the anthracycline-related structural changes that have been well established ^27, 28^. When trastuzumab is combined with anthracycline therapy, the cardiotoxicity has been shown to be profound and noted in over 25% of the patients treated with both agents ^29, 30^. When used without anthracyclines, trastuzumab is associated with HF in about 2–4% of patients ^26, 31^.

The mechanism of cardiac toxicity from trastuzumab is related to selective targeting of tyrosine kinases that have both “on-target” and “off target” effects on the heart. Inhibition of ErbB2/HER2 and subsequently reduced ErbB signaling in cancer cells results in cell-mediated cytotoxicity, the major proposed mechanism of trastuzumab’s therapeutic effect for breast cancers with over-expression of this pro-survival pathway. However, ErbB is one of the aforementioned pro-survival signaling pathways in cardiomyocytes that are possibly affected by anthracyclines and this “on-target” inhibition is speculated to be the major mechanism of cardiac toxicity. Supporting this theory, mutation or deletion of HER2 is known to be associated with dilated cardiomyopathy in mice with exaggerated sensitivity to pressure overload ^32, 33^. The more favorable cardiac safety profiles of two other HER2 inhibitors—lapatinib and pertuzumab—have raised questions of additional “off-target” effects of trastuzumab-mediated toxicity, although the mechanisms are less well characterized ^34– 38^.

Although the cardiac toxicity of trastuzumab is generally considered to be reversible with discontinuation of therapy, the role for guideline-directed medical therapy for left ventricular dysfunction such as β-blockers or ACE inhibitors is less well established. The use of these agents and other preventative strategies, such as exercise, will be discussed in the second part of this review series.

### Immune checkpoint inhibitors

Remarkable progress has been made in the management of several malignancies, including advanced melanoma, with the use of agents that inhibit immune checkpoint mediators. The first two agents in this class—ipilimumab and nivolumab—have dramatically altered the landscape of antineoplastic therapy but have also been associated with autoimmune adverse reactions ^39, 40^. By allowing the immune system to increase its activity against malignant cells, checkpoint inhibitors cause patients to become more vulnerable to attacks on healthy tissue, causing autoimmune complications such as hepatitis, pneumonitis, and adrenal insufficiency ^41, 42^. Recently, cardiotoxicity in the form of potentially fatal myocarditis has been reported, especially with ipilimumab and nivolumab combination therapy ^43^. At least 15 cases of cardiotoxicity related to immune checkpoint inhibitors have been reported with manifestations including myocarditis and complete heart block, although these numbers likely underestimate the true incidence ^44, 45^.

Based on biopsies from tumor as well as heart and skeletal muscle in two patients who experienced fulminant myocarditis, Johnson *et al*. suggested that activated T cells may be targeting an antigen shared by the tumor and myocytes in skeletal and cardiac muscle. This “on-target” effect ultimately results in autoimmune myocarditis and myositis ^43^. In the inflammatory state, it also appears that PD-L1, which was expressed on the injured myocytes, is protective and its inhibition by these agents contributes to cardiomyocyte vulnerability to injury ^46^. Although few guidelines exist on the management of these complications, the second part of this review series will discuss considerations for prevention, monitoring, and treatment of immunotherapy-related cardiotoxicity.

### Vascular endothelial growth factor inhibitors

Inhibitors of the vascular endothelial growth factor (VEGF) pathway such as sunitinib and sorafenib are used to treat a variety of malignancies, including renal cell carcinoma and hepatocellular carcinoma. Similar to the other agents discussed above, they are highly effective but often limited by their cardiovascular toxicity, especially hypertension and left ventricular dysfunction. Hypertension has been reported in nearly half of the patients receiving VEGF inhibitors, which can be of varying clinical significance depending on the baseline blood pressure and comorbid conditions ^47– 49^. Asymptomatic decline in left ventricular function is noted in approximately a quarter of patients, whereas symptomatic HF is seen in 4–8% ^47, 50^.

VEGF promotes the growth and development of blood vessels by stimulating tyrosine kinase receptors on the cell surface, a process essential to the growth of various malignancies. The therapeutic mechanism of action of VEGF inhibitors is to bind and inhibit the ATP binding site of these specific kinases, thereby inhibiting the angiogenesis. These agents have been shown *in vitro* to inhibit doses of kinases, and cardiovascular toxicities from these agents occur through inhibition of both “on-target” and “off-target” kinases ^51^.

A variety of mechanisms have been proposed for the hypertension seen with VEGF inhibitors. Endothelin-1, which is a potent vasoconstrictor, is increased with VEGF inhibition, and endothelin antagonism appears to limit the development of hypertension, although the mechanism by which VEGF inhibitors activate endothelin-1 has not been clearly established ^52, 53^. Increasing evidence suggests that VEGF activates the production and release of nitric oxide, a potent vasodilator, although experimental evidence has not conclusively shown decreased nitric oxide bioavailability with VEGF inhibition ^54– 56^. The inhibition of angiogenesis in the target malignancy causes some degree of microcapillary rarefaction to occur and with this the peripheral vascular resistance and subsequently the blood pressure would be expected to rise. The clinical significance of this mechanism is very unclear both because of the degree of reduction in capillary density that would be necessary to achieve a significant increase in blood pressure and because the hypertension from VEGF inhibitors has been shown to occur rapidly, usually within hours, whereas microcapillary rarefaction appears to take days to weeks to occur ^52, 57– 59^.

It is likely that the consistent and often marked hypertension that occurs contributes to cardiac dysfunction through increased afterload on the left ventricle ^18^. Additionally, inhibition of several kinases has been proposed to contribute to left ventricular dysfunction, although the evidence relies largely on non-human models of unclear clinical significance. Alterations in the activity of AMPK, a kinase which is critical in regulating myocardial energetics and mitochondrial function, are inhibited especially by sunitinib, although the significance of this in humans has not been established ^60, 61^. ERK, another kinase that is inhibited by sorafenib, is important for cardioprotection with increased stress, which may be especially important in the setting of increased afterload from drug-related systemic hypertension ^62, 63^. Pressure overload in the setting of ERK inhibition may promote apoptosis of cardiomyocytes ^64, 65^. Some degree of inhibition of many other kinases has been hypothesized to play a role in the development of cardiotoxicity, although the clinical significance of these has not been established. Management of the hypertension and left ventricular dysfunction will be discussed in the second part of this review series.

## Conclusions

Both traditional chemotherapeutic agents and newer therapies have important effects on myocardial tissues resulting in arrhythmias, HF, and death. The mechanism of these toxicities consistently relates to “on-target” therapeutic mechanisms of the antineoplastic agents, suggesting a possibly inescapable link between these life-saving medications and cardiovascular toxicity. As new therapies are developed and especially as they are used in aging populations with diminished cardiac reserve, it is essential that oncologists collaborate closely with cardiologists to monitor for and manage these complications. It is also essential to recognize the multifactorial processes that lead to the development of cardiovascular disease in patients treated for malignancies. In the next installment of this review series, we will discuss the management of cardiac toxicity and how these strategies relate to the proposed mechanisms discussed here.

## References

[1] Murphy SL Xu J Kochanek KD: Deaths: final data for 2010. *Natl Vital Stat Rep.* 2013;61(4):1–117. 24979972

[2] Oeffinger KC Mertens AC Sklar CA: Chronic health conditions in adult survivors of childhood cancer. *N Engl J Med.* 2006;355(15):1572–82. 10.1056/NEJMsa060185 17035650

[3] Lipshultz SE Franco VI Miller TL: Cardiovascular disease in adult survivors of childhood cancer. *Annu Rev Med.* 2015;66:161–76. 10.1146/annurev-med-070213-054849 25587648 PMC5057395

[4] Çetin S Babaoğlu K Başar EZ: Subclinical anthracycline-induced cardiotoxicity in long-term follow-up of asymptomatic childhood cancer survivors: Assessment by speckle tracking echocardiography. *Echocardiography.* 2017. 10.1111/echo.13743 29106752

[5] Hequet O Le QH Moullet I: Subclinical late cardiomyopathy after doxorubicin therapy for lymphoma in adults. *J Clin Oncol.* 2004;22(10):1864–71. 10.1200/JCO.2004.06.033 15143078

[6] Chang H Okwuosa TM Scarabelli T: Cardiovascular Complications of Cancer Therapy: Best Practices in Diagnosis, Prevention, and Management: Part 2. *J Am Coll Cardiol.* 2017;70(20):2552–65. 10.1016/j.jacc.2017.09.1095 29145955 PMC5825188

[7] Enriquez A Biagi J Redfearn D: Increased Incidence of Ventricular Arrhythmias in Patients With Advanced Cancer and Implantable Cardioverter-Defibrillators. *JACC Clin Electrophysiol.* 2017;3(1):50–6. 10.1016/j.jacep.2016.03.001 29759695

[8] Broder H Gottlieb RA Lepor NE: Chemotherapy and cardiotoxicity. *Rev Cardiovasc Med.* 2008;9(2):75–83. 18660728 PMC3723407

[9] Markman TM Nazarian S: Arrhythmia and Electrophysiological Effects of Chemotherapy: A Review. *Oncology.* 2016;91(2):61–8. 10.1159/000446374 27256307

[10] Cardinale D Colombo A Lamantia G: Anthracycline-induced cardiomyopathy: clinical relevance and response to pharmacologic therapy. *J Am Coll Cardiol.* 2010;55(3):213–20. 10.1016/j.jacc.2009.03.095 20117401

[11] Volkova M Russell R 3rd: Anthracycline cardiotoxicity: prevalence, pathogenesis and treatment. *Curr Cardiol Rev.* 2011;7(4):214–20. 10.2174/157340311799960645 22758622 PMC3322439

[12] Swain SM Whaley FS Ewer MS: Congestive heart failure in patients treated with doxorubicin: a retrospective analysis of three trials. *Cancer.* 2003;97(11):2869–79. 10.1002/cncr.11407 12767102

[13] Simůnek T Stérba M Popelová O: Anthracycline-induced cardiotoxicity: overview of studies examining the roles of oxidative stress and free cellular iron. *Pharmacol Rep.* 2009;61(1):154–71. 10.1016/S1734-1140(09)70018-0 19307704

[14] Jones RL Swanton C Ewer MS: Anthracycline cardiotoxicity. *Expert Opin Drug Saf.* 2006;5(6):791–809. 10.1517/14740338.5.6.791 17044806

[15] Rahman AM Yusuf SW Ewer MS: Anthracycline-induced cardiotoxicity and the cardiac-sparing effect of liposomal formulation. *Int J Nanomedicine.* 2007;2(4):567–83. 18203425 PMC2676818

[16] Markman TM Markman M: Cardiotoxicity of antineoplastic agents: what is the present and future role for imaging? *Curr Oncol Rep.* 2014;16(8):396. 10.1007/s11912-014-0396-y 24992733

[17] Markman TM Ruble K Loeb D: Electrophysiological effects of anthracyclines in adult survivors of pediatric malignancy. *Pediatr Blood Cancer.* 2017;64(11):e26556. 10.1002/pbc.26556 28453898

[18] Hahn VS Lenihan DJ Ky B: Cancer therapy-induced cardiotoxicity: basic mechanisms and potential cardioprotective therapies. *J Am Heart Assoc.* 2014;3(2):e000665. 10.1161/JAHA.113.000665 24755151 PMC4187516

[19] Chen Z Norris JY Finck BN: Peroxisome proliferator-activated receptor-gamma coactivator-1alpha (PGC-1alpha) stimulates VLDL assembly through activation of cell death-inducing DFFA-like effector B (CideB). *J Biol Chem.* 2010;285(34):25996–6004. 10.1074/jbc.M110.141598 20551328 PMC2923994

[20] Finck BN Kelly DP: Peroxisome proliferator-activated receptor gamma coactivator-1 (PGC-1) regulatory cascade in cardiac physiology and disease. *Circulation.* 2007;115(19):2540–8. 10.1161/CIRCULATIONAHA.107.670588 17502589

[21] De Angelis A Piegari E Cappetta D: Anthracycline cardiomyopathy is mediated by depletion of the cardiac stem cell pool and is rescued by restoration of progenitor cell function. *Circulation.* 2010;121(2):276–92. 10.1161/CIRCULATIONAHA.109.895771 20038740 PMC2810713

[22] Huang C Zhang X Ramil JM: Juvenile exposure to anthracyclines impairs cardiac progenitor cell function and vascularization resulting in greater susceptibility to stress-induced myocardial injury in adult mice. *Circulation.* 2010;121(5):675–83. 10.1161/CIRCULATIONAHA.109.902221 20100968 PMC2834271

[23] Horie T Ono K Nishi H: Acute doxorubicin cardiotoxicity is associated with miR-146a-induced inhibition of the neuregulin-ErbB pathway. *Cardiovasc Res.* 2010;87(4):656–64. 10.1093/cvr/cvq148 20495188 PMC2920811

[24] Liu X Gu X Li Z: Neuregulin-1/erbB-activation improves cardiac function and survival in models of ischemic, dilated, and viral cardiomyopathy. *J Am Coll Cardiol.* 2006;48(7):1438–47. 10.1016/j.jacc.2006.05.057 17010808

[25] Maurea N Coppola C Ragone G: Women survive breast cancer but fall victim to heart failure: the shadows and lights of targeted therapy. *J Cardiovasc Med (Hagerstown).* 2010;11(12):861–8. 10.2459/JCM.0b013e328336b4c1 20072001

[26] Hudis CA: Trastuzumab--mechanism of action and use in clinical practice. *N Engl J Med.* 2007;357(1):39–51. 10.1056/NEJMra043186 17611206

[27] Ewer SM Ewer MS: Cardiotoxicity profile of trastuzumab. *Drug Saf.* 2008;31(6):459–67. 10.2165/00002018-200831060-00002 18484781

[28] Ewer MS Vooletich MT Durand JB: Reversibility of trastuzumab-related cardiotoxicity: new insights based on clinical course and response to medical treatment. *J Clin Oncol.* 2005;23(31):7820–6. 10.1200/JCO.2005.13.300 16258084

[29] Slamon D Eiermann W Robert N: Adjuvant trastuzumab in HER2-positive breast cancer. *N Engl J Med.* 2011;365(14):1273–83. 10.1056/NEJMoa0910383 21991949 PMC3268553

[30] Bowles EJ Wellman R Feigelson HS: Risk of heart failure in breast cancer patients after anthracycline and trastuzumab treatment: a retrospective cohort study. *J Natl Cancer Inst.* 2012;104(17):1293–305. 10.1093/jnci/djs317 22949432 PMC3433392

[31] Chang HM Moudgil R Scarabelli T: Cardiovascular Complications of Cancer Therapy: Best Practices in Diagnosis, Prevention, and Management: Part 1. *J Am Coll Cardiol.* 2017;70(20):2536–51. 10.1016/j.jacc.2017.09.1096 29145954 PMC5825187

[32] Bria E Cuppone F Milella M: Trastuzumab cardiotoxicity: biological hypotheses and clinical open issues. *Expert Opin Biol Ther.* 2008;8(12):1963–71. 10.1517/14728220802517935 18990083

[33] Ozcelik C Erdmann B Pilz B: Conditional mutation of the ErbB2 (HER2) receptor in cardiomyocytes leads to dilated cardiomyopathy. *Proc Natl Acad Sci U S A.* 2002;99(13):8880–5. 10.1073/pnas.122249299 12072561 PMC124392

[34] Force T Krause DS Van Etten RA: Molecular mechanisms of cardiotoxicity of tyrosine kinase inhibition. *Nat Rev Cancer.* 2007;7(5):332–44. 10.1038/nrc2106 17457301

[35] Li W Croce K Steensma DP: Vascular and Metabolic Implications of Novel Targeted Cancer Therapies: Focus on Kinase Inhibitors. *J Am Coll Cardiol.* 2015;66(10):1160–78. 10.1016/j.jacc.2015.07.025 26337996

[36] Perez EA Barrios C Eiermann W: Trastuzumab Emtansine With or Without Pertuzumab Versus Trastuzumab Plus Taxane for Human Epidermal Growth Factor Receptor 2-Positive, Advanced Breast Cancer: Primary Results From the Phase III MARIANNE Study. *J Clin Oncol.* 2017;35(2):141–8. 10.1200/JCO.2016.67.4887 28056202 PMC5455677

[37] Perez EA Koehler M Byrne J: Cardiac safety of lapatinib: pooled analysis of 3689 patients enrolled in clinical trials. *Mayo Clin Proc.* 2008;83(6):679–86. 10.4065/83.6.679 18533085

[38] Swain SM Ewer MS Cortés J: Cardiac tolerability of pertuzumab plus trastuzumab plus docetaxel in patients with HER2-positive metastatic breast cancer in CLEOPATRA: a randomized, double-blind, placebo-controlled phase III study. *Oncologist.* 2013;18(3):257–64. 10.1634/theoncologist.2012-0448 23475636 PMC3607520

[39] Naidoo J Page DB Li BT: Toxicities of the anti-PD-1 and anti-PD-L1 immune checkpoint antibodies. *Ann Oncol.* 2015;26(12):2375–91. 10.1093/annonc/mdv383 26371282 PMC6267867

[40] Champiat S Lambotte O Barreau E: Management of immune checkpoint blockade dysimmune toxicities: a collaborative position paper. *Ann Oncol.* 2016;27(4):559–74. 10.1093/annonc/mdv623 26715621

[41] Weber JS Kähler KC Hauschild A: Management of immune-related adverse events and kinetics of response with ipilimumab. *J Clin Oncol.* 2012;30(21):2691–7. 10.1200/JCO.2012.41.6750 22614989

[42] Nishino M Giobbie-Hurder A Hatabu H: Incidence of Programmed Cell Death 1 Inhibitor-Related Pneumonitis in Patients With Advanced Cancer: A Systematic Review and Meta-analysis. *JAMA Oncol.* 2016;2(12):1607–16. 10.1001/jamaoncol.2016.2453 27540850

[43] Johnson DB Balko JM Compton ML: Fulminant Myocarditis with Combination Immune Checkpoint Blockade. *N Engl J Med.* 2016;375(18):1749–55. 10.1056/NEJMoa1609214 27806233 PMC5247797

[44] Jain V Bahia J Mohebtash M: Cardiovascular Complications Associated With Novel Cancer Immunotherapies. *Curr Treat Options Cardiovasc Med.* 2017;19(5):36. 10.1007/s11936-017-0532-8 28401456

[45] Behling J Kaes J Münzel T: New-onset third-degree atrioventricular block because of autoimmune-induced myositis under treatment with anti-programmed cell death-1 (nivolumab) for metastatic melanoma. *Melanoma Res.* 2017;27(2):155–8. 10.1097/CMR.0000000000000314 27977496

[46] Cheng F Loscalzo J: Autoimmune Cardiotoxicity of Cancer Immunotherapy. *Trends Immunol.* 2017;38(2):77–8. 10.1016/j.it.2016.11.007 27919707

[47] Chu TF Rupnick MA Kerkela R: Cardiotoxicity associated with tyrosine kinase inhibitor sunitinib. *Lancet.* 2007;370(9604):2011–9. 10.1016/S0140-6736(07)61865-0 18083403 PMC2643085

[48] Zhu X Stergiopoulos K Wu S: Risk of hypertension and renal dysfunction with an angiogenesis inhibitor sunitinib: systematic review and meta-analysis. *Acta Oncol.* 2009;48(1):9–17. 10.1080/02841860802314720 18752081

[49] Wu S Chen JJ Kudelka A: Incidence and risk of hypertension with sorafenib in patients with cancer: a systematic review and meta-analysis. *Lancet Oncol.* 2008;9(12):117–23. 10.1016/S1470-2045(08)70003-2 18221915

[50] Richards CJ Je Y Schutz FA: Incidence and risk of congestive heart failure in patients with renal and nonrenal cell carcinoma treated with sunitinib. *J Clin Oncol.* 2011;29(25):3450–6. 10.1200/JCO.2010.34.4309 21810682

[51] Ghoreschi K Laurence A O'Shea JJ: Selectivity and therapeutic inhibition of kinases: to be or not to be? *Nat Immunol.* 2009;10(4):356–60. 10.1038/ni.1701 19295632 PMC2758543

[52] Kappers MH van Esch JH Sluiter W: Hypertension induced by the tyrosine kinase inhibitor sunitinib is associated with increased circulating endothelin-1 levels. *Hypertension.* 2010;56(4):675–81. 10.1161/HYPERTENSIONAHA.109.149690 20733093

[53] Kappers MH Smedts FM Horn T: The vascular endothelial growth factor receptor inhibitor sunitinib causes a preeclampsia-like syndrome with activation of the endothelin system. *Hypertension.* 2011;58(2):295–302. 10.1161/HYPERTENSIONAHA.111.173559 21670421

[54] Hood JD Meininger CJ Ziche M: VEGF upregulates ecNOS message, protein, and NO production in human endothelial cells. *Am J Physiol.* 1998;274(3 Pt 2):H1054–8. 10.1152/ajpheart.1998.274.3.H1054 9530221

[55] Robinson ES Khankin EV Choueiri TK: Suppression of the nitric oxide pathway in metastatic renal cell carcinoma patients receiving vascular endothelial growth factor-signaling inhibitors. *Hypertension.* 2010;56(6):1131–6. 10.1161/HYPERTENSIONAHA.110.160481 20956731 PMC3078049

[56] Mayer EL Dallabrida SM Rupnick MA: Contrary effects of the receptor tyrosine kinase inhibitor vandetanib on constitutive and flow-stimulated nitric oxide elaboration in humans. *Hypertension.* 2011;58(1):85–92. 10.1161/HYPERTENSIONAHA.110.168120 21482957 PMC4586117

[57] Baffert F Le T Sennino B: Cellular changes in normal blood capillaries undergoing regression after inhibition of VEGF signaling. *Am J Physiol Heart Circ Physiol.* 2006;290(2):H547–59. 10.1152/ajpheart.00616.2005 16172161

[58] Steeghs N Gelderblom H Roodt JO: Hypertension and rarefaction during treatment with telatinib, a small molecule angiogenesis inhibitor. *Clin Cancer Res.* 2008;14(11):3470–6. 10.1158/1078-0432.CCR-07-5050 18519779

[59] de Jesus-Gonzalez N Robinson E Moslehi J: Management of antiangiogenic therapy-induced hypertension. *Hypertension.* 2012;60(3):607–15. 10.1161/HYPERTENSIONAHA.112.196774 22851729 PMC3421063

[60] Hasinoff BB Patel D O'Hara KA: Mechanisms of myocyte cytotoxicity induced by the multiple receptor tyrosine kinase inhibitor sunitinib. *Mol Pharmacol.* 2008;74(6):1722–8. 10.1124/mol.108.050104 18815214

[61] Kerkela R Woulfe KC Durand JB: Sunitinib-induced cardiotoxicity is mediated by off-target inhibition of AMP-activated protein kinase. *Clin Transl Sci.* 2009;2(1):15–25. 10.1111/j.1752-8062.2008.00090.x 20376335 PMC2849142

[62] Harris IS Zhang S Treskov I: Raf-1 kinase is required for cardiac hypertrophy and cardiomyocyte survival in response to pressure overload. *Circulation.* 2004;110(6):718–23. 10.1161/01.CIR.0000138190.50127.6A 15289381

[63] Chen J Fujii K Zhang L: Raf-1 promotes cell survival by antagonizing apoptosis signal-regulating kinase 1 through a MEK-ERK independent mechanism. *Proc Natl Acad Sci U S A.* 2001;98(14):7783–8. 10.1073/pnas.141224398 11427728 PMC35419

[64] Cheng H Kari G Dicker AP: A novel preclinical strategy for identifying cardiotoxic kinase inhibitors and mechanisms of cardiotoxicity. *Circ Res.* 2011;109(12):1401–9. 10.1161/CIRCRESAHA.111.255695 21998323 PMC3908774

[65] Yamaguchi O Watanabe T Nishida K: Cardiac-specific disruption of the *c-raf-1* gene induces cardiac dysfunction and apoptosis. *J Clin Invest.* 2004;114(7):937–43. 10.1172/JCI20317 15467832 PMC518660

